# Clinical outcomes of empirical anti-MRSA therapy at a tertiary care center in Madinah, Saudi Arabia: a retrospective single-center cohort study

**DOI:** 10.1186/s12879-026-13283-w

**Published:** 2026-04-16

**Authors:** Fatimah Aljohani, Khadiga Suliman, Alaa F. Alsehemi, Elaf Kinani, Roaa Alrehaili, Lamar Alkadi, Hanan Alshareef

**Affiliations:** 1 Pharmacy Department, Prince Sultan Armed Forces Hospital, P.O. 42375, Mohamed Bin Sohail Street, Defense District of Medina, Medina, Saudi Arabia; 2 Pharmacy Department, Al-Thager Hospital, Ministry of Health, P.O. 22361, Jeddah, Saudi Arabia; 3 College of Pharmacy, University of Hail, P.O. 55473, Medina, Saudi Arabia; 4 Pharmacy Practice Department, Faculty of Pharmacy, University of Tabuk, P.O. 47713, Tabuk, Saudi Arabia

**Keywords:** MRSA, Antibiotics, Lenzolid, Vancomycin, In-hospital mortality

## Abstract

**Background:**

Methicillin-resistant *Staphylococcus aureus* (MRSA) is highly prevalent in Saudi Arabia, posing a significant clinical challenge. International guidelines recommend empirical anti-MRSA coverage for high-risk patients. This study aimed to describe the real-world use of empirical anti-MRSA therapies and explore associated clinical outcomes among hospitalized adults at a single tertiary center in Saudi Arabia.

**Methods:**

We conducted a retrospective cohort study of 323 adult patients who received empirical intravenous anti-MRSA therapy (linezolid, vancomycin, or teicoplanin) between June 2021 and June 2025. The primary endpoint was in-hospital mortality. Secondary endpoints included clinical improvement, length of stay, adverse events, and changes in laboratory measures. Multivariable logistic regression was used to identify independent predictors of mortality.

**Results:**

The cohort had a mean age of 70.97 years, and 51.70% had started empirical anti-MRSA during ICU stay. Overall, the in-hospital mortality rate was 39.01%. Mortality rates did not differ significantly between treatment groups: 42.57% for linezolid, 35.82% for vancomycin, and 57.89% for teicoplanin (*p* = 0.121). Changes in C-reactive protein varied significantly (*p* = 0.009), with levels decreasing in the linezolid and vancomycin groups but increasing in the small teicoplanin group. Vancomycin was associated with a higher reported incidence of acute kidney injury than linezolid and teicoplanin (3.00% vs. 0.99% and 0.00%, respectively). In multivariable analysis, older age (OR 1.05, *p* < 0.001) and male sex (OR 1.81, *p* = 0.032) were significant risk factors for mortality. Non-ICU admission was associated with a lower mortality rate (OR 0.45, *p* = 0.004).

**Conclusion:**

In this real-world cohort of patients receiving empirical anti-MRSA therapy, we found no significant difference in in-hospital mortality or clinical improvement between linezolid, vancomycin, and teicoplanin. Patient outcomes were primarily driven by underlying factors like age and illness severity rather than the choice of antibiotics. Vancomycin was associated with a higher incidence of reported acute kidney injury. The findings highlight the need for improved antimicrobial stewardship to guide appropriate empirical therapy.

## Introduction

Methicillin-resistant *Staphylococcus aureus* (MRSA) represents a formidable global health threat, imposing a significant burden of morbidity, mortality, and healthcare expenditure, particularly within hospital settings [1]. In the Middle East and specifically in Saudi Arabia, MRSA infections are a pressing clinical issue. The national prevalence of MRSA in Saudi Arabia is estimated to be as high as 38%, with considerable regional variation; the Western region reports rates exceeding 40%, potentially influenced by the annual congregation of millions for religious pilgrimages [2, 3]. This high prevalence is associated with severe clinical consequences, including mortality rates for hospital-acquired MRSA pneumonia estimated between 30% and 35% [4]. The widespread dissemination of hospital-acquired (HA-MRSA) strains such as ST239-III further complicates the epidemiological landscape in the region [5].

Given the severe outcomes associated with delayed or inappropriate treatment of MRSA infections, international guidelines, including those from the Surviving Sepsis Campaign, advocate for empirical anti-MRSA antibiotic coverage in critically ill patients with sepsis or septic shock who possess risk factors for MRSA [6]. These risk factors commonly include recent hospitalization, prior intravenous antibiotic use, the presence of invasive devices, or known MRSA colonization [7]. The rationale for this aggressive initial approach is to mitigate the increased mortality associated with MRSA bacteremia when effective therapy is not promptly initiated [8].

However, the strategy of broad empirical anti-MRSA coverage is not without significant controversy. The central debate questions whether this approach provides a net clinical benefit or contributes to unintended harm. A large retrospective cohort study involving over 88,000 pneumonia patients found that empirical anti-MRSA treatment was not associated with improved survival and, in some patient subgroups, was linked to an increased adjusted risk of death, acute kidney injury (AKI), and secondary infections like *Clostridioides difficile* [8]. Such findings highlight the critical need for robust antimicrobial stewardship and a nuanced approach guided by local epidemiology and individual patient risk assessment, rather than a universal protocol [7, 9].

Selecting between vancomycin and linezolid for empirical anti-MRSA coverage adds further complexity to clinical decision-making. Vancomycin has long been the standard of care, but concerns regarding its pharmacokinetic variability, potential for nephrotoxicity, and suboptimal penetration into certain tissues like the lung epithelium persist [10]. In contrast, some studies suggest linezolid may offer improved outcomes, including lower mortality in patients with MRSA pneumonia, potentially due to its superior lung tissue penetration [11]. However, meta-analyses have yielded conflicting results, with several large reviews finding no significant difference in all-cause mortality between the two agents for MRSA infections [12, 13]. Regarding safety, linezolid has been consistently associated with a lower incidence of nephrotoxicity compared to vancomycin in several head-to-head comparisons [10, 14]. This leaves clinicians with an unresolved dilemma, balancing conflicting efficacy data against a clearer safety advantage, with a notable gap in evidence from regions like Saudi Arabia, where local resistance patterns and patient populations may influence outcomes.

Given the high prevalence of MRSA in Saudi Arabia and the ongoing global debate regarding the risks and benefits of empirical coverage, there is a critical need for local data to guide clinical practice. Therefore, the objective of this study was to describe the use of empirical anti-MRSA therapy in routine clinical practice and to explore associated clinical outcomes, including mortality and adverse events, among hospitalized patients at a single tertiary care center in Saudi Arabia.

## Methods

This study was conducted and reported in accordance with the Strengthening the Reporting of Observational Studies in Epidemiology (STROBE) guidelines for cohort studies. This study was conducted in accordance with the ethical principles of the Declaration of Helsinki.

### Study design and setting

This was a retrospective, observational cohort study conducted at Prince Sultan Armed Forces Hospital, a tertiary care facility in Madinah, Saudi Arabia. The study period extended from June 2021 until June 2025. The Prince Sultan Armed Forces Hospital’s institutional review board granted ethical approval for the study (IRB: REC-25-13) and waived the requirement for informed consent due to the retrospective nature of the data analysis.

### Study population

The study included adult inpatients who were admitted to the ICU, medical wards, or surgical wards and received empirical therapy for suspected MRSA infection. No formal a priori sample size calculation was performed because this was a retrospective cohort study, and the study size was determined by including all consecutive eligible patients identified during the predefined study period from the hospital’s electronic records.

### Eligibility criteria

Patients were included if they met all of the following criteria:

1. Aged 18 years or older.
2. Admitted to Intensive Care Unit (ICU), medical wards, or surgical wards.
3. Received empirical intravenous (IV) anti-MRSA therapy (vancomycin, linezolid, or teicoplanin) for at least 24 h, to distinguish initiated empirical treatment from transient or incidental exposure.
4. Had complete electronic medical records (EMRs) available for the duration of their hospital stay.

Patients were excluded if they met any of the following criteria:

1. Age less than 18 years.
2. Confirmed MRSA infection prior to starting MRSA therapy.
3. Receipt of oral vancomycin only (e.g., for *Clostridioides difficile* infection).
4. Incomplete or missing data pertinent to primary or key secondary outcomes.

### Data collection and variables

Data were retrospectively extracted from the hospital’s EMR using a standardized data collection form. For each patient, we collected demographics (age, sex, height, weight, and body mass index [BMI]); clinical characteristics (ward of admission, comorbidities, infection type, such as sepsis or pneumonia, and the indication for empirical MRSA coverage); microbiological findings (results of blood, respiratory, or other relevant cultures); treatment details (the empirical specific anti-MRSA agent prescribed, initial dose, dosing frequency, and total duration of therapy); and laboratory data. Laboratory variables included baseline and follow-up measures of renal function (creatinine clearance), liver function (alanine aminotransferase [ALT] and aspartate aminotransferase [AST]), complete blood count (white blood cell and platelet counts), and inflammatory markers (C-reactive protein [CRP] and temperature). Each patient was followed from the initiation of empirical anti-MRSA therapy until hospital discharge or in-hospital death.

### Outcomes

The primary endpoint was in-hospital mortality, defined as death from any cause during the index admission. Secondary endpoints comprised clinical improvement (i.e., infection resolution), length of hospital stay (total days from admission to discharge or death), and any documented adverse drug events, including AKI, elevated liver enzymes, and gastrointestinal upset. For the purpose of this retrospective study, clinical improvement (infection resolution) was defined as documented improvement or resolution of the suspected index infection in the medical record by the end of empirical anti-MRSA therapy or at hospital discharge, without documented escalation of anti-MRSA therapy due to persistent clinical deterioration. This assessment was supported by improvement in available clinical and laboratory parameters, where applicable, including temperature, white blood cell count, and C-reactive protein. Length of hospital stay was defined as the total number of days from hospital admission to discharge or death. No post-discharge follow-up data were available; therefore, mortality was assessed only during the index hospitalization. We also evaluated changes in clinical and laboratory parameters from baseline to the end of therapy, specifically temperature, creatinine clearance, ALT, AST, CRP, white blood cell count, and platelet count. In addition, potential risk factors associated with in-hospital mortality were explored using univariate and multivariable logistic regression analyses, including demographic, clinical, and laboratory variables such as age, sex, BMI, ICU admission, comorbidities, infection type, and baseline renal and hepatic function.

### Statistical analysis

All statistical analyses were conducted using Jamovi Software (Windows version 2.6.23). Baseline demographic and clinical characteristics were summarized descriptively: continuous variables are reported as mean ± standard deviation (SD) when approximately normally distributed or as median with interquartile range (IQR) when skewed; categorical variables are presented as counts and percentages (n, %). Between-group comparisons were performed across the empirical treatment cohorts (e.g., vancomycin, linezolid, and teicoplanin). Categorical variables were compared using Pearson’s χ² test or Fisher’s exact test when expected cell counts were small. Continuous variables were compared using one-way ANOVA for normally distributed data (with homogeneity of variances assessed) or the Kruskal–Wallis test for non-normally distributed data; where only two groups were contrasted, Student’s t test or the Mann–Whitney U test was used as appropriate. Where relevant, 95% confidence intervals were reported to convey the precision of estimates. To identify predictors of in-hospital mortality, we first ran univariate logistic regressions for prespecified covariates. Variables with clinical plausibility and/or a univariate association at *p* < 0.05 were entered into a multivariable logistic regression using a purposeful selection approach; results are presented as odds ratios (ORs) with 95% confidence intervals (CIs) and two-sided p values. Time-to-event analyses were conducted with Cox proportional hazards models, defining time zero as the start of empirical anti-MRSA therapy and censoring at hospital discharge; results are reported as hazard ratios (HRs) with 95% CIs. Kaplan–Meier survival curves were generated for the overall cohort and stratified by key subgroups (sex, age group, ICU admission, initial antibiotic, BMI category), with group differences compared using the log-rank test. Curves display 95% CIs and number-at-risk tables. All tests were two-sided with α = 0.05; no formal multiplicity adjustment was applied, so secondary and subgroup findings should be interpreted as exploratory. Because this was a retrospective study based on routinely collected EMR data, no formal imputation procedures were performed. Analyses were conducted using available-case data, and missing or undocumented values were reported explicitly where applicable.

## Results

### Demographic and clinical characteristics

The study cohort had a mean age of 70.97 ± 17.26 years, with a slight male predominance (53.25% male vs. 46.75% female). The mean height and weight were 158.43 ± 9.96 cm and 70.71 ± 19.11 kg, respectively, yielding a mean BMI of 28.28 ± 8.01 kg/m². Most patients had been starting the empirical anti- MRSA therapy during ICU admission (51.70%), followed by medical wards (27.55%), and surgical wards (20.12%). Regarding microbiology, 43.96% of cultures showed no growth, while Gram-positive cocci (27.24%) and Gram-negative bacilli (25.39%) were commonly identified. Among all isolates, MRSA was reported in 9.29% of cases. The most frequent organisms isolated were Klebsiella pneumoniae (17.03%), Staphylococcus aureus (9.29%), and Staphylococcus *epidermidis* (4.33%), as shown in Table 1.

Empirical MRSA coverage was most commonly indicated due to immunosuppression or comorbidities (46.44%), with other contributing factors including recent hospitalization (21.67%), severe infection (11.46%), and recent intravenous antibiotics (3.72%). The median hospital length of stay was 16 days (IQR: 9–30). The most frequently prescribed anti-MRSA were vancomycin (62.54%), linezolid (31.58%), and teicoplanin (5.88%). The median antibiotic course duration was 5 days (IQR: 3–8). Regarding infection types, the most frequent were sepsis (25.08%), septic shock (19.50%), and COVID-19 pneumonia (16.72%).

Across treatment groups, baseline age and BMI were comparable (*p* = 0.368 and *p* = 0.603), respectively. Female patients were more frequent in the linezolid group (55.88%) than in the vancomycin (43.56%) and teicoplanin (31.58%) groups. Ward of admission differed significantly between treatment groups (*p* < 0.001); vancomycin was more commonly used in medical wards, whereas linezolid and teicoplanin were more frequently prescribed in surgical wards, while approximately half of patients in each group were admitted to the ICU. The proportion of patients admitted to the ICU remained comparable across all treatment groups (approximately 47–53%). Culture categories were broadly similar (*p* = 0.587), and treatment duration did not differ significantly (median [IQR] days: 5 [3–6] for linezolid, 5 [3–8] for vancomycin, 6 [3–9] for teicoplanin; *p* = 0.448), as shown in Table 1.

Indications for MRSA coverage showed significant between-group differences (*p* < 0.001). Immunosuppression or multiple comorbidities were the most common indications for initiating empirical linezolid (62.8%), compared with vancomycin (39.1%) and teicoplanin (36.8%). Recent hospitalization within the preceding 90 days was also a frequent reason for starting therapy, particularly among patients receiving teicoplanin (47.4%) and linezolid (30.4%), compared with those who received vancomycin (14.9%). The distribution of infection categories was similar across treatment groups (*p* = 0.486). Bloodstream/sepsis-related infections were the most common category among patients receiving linezolid (47.06%) and vancomycin (45.05%), whereas respiratory tract infections predominated among those receiving teicoplanin (57.89%).

**Table 1:** Baseline demographic and clinical characteristics of patients by treatment group

Variables		Total	Linezolid	Vancomycin	Teicoplanin	*P*-value
Age, years	Mean ± SD	70.97 ± 17.26	72.13 ± 15.59	70.04 ± 18.18	74.58 ± 15.60	0.368
Gender, n (%)	Male	172 (53.25%)	45 (44.12%)	114 (56.44%)	13 (68.42%)	0.050
	Female	151 (46.75%)	57 (55.88%)	88 (43.56%)	6 (31.58%)	
BMI, Kg/m^2^	Mean ± SD	28.28 ± 8.01	29.13 ± 9.76	27.93 ± 7.17	27.83 ± 7.03	0.603
Ward of Admission, n (%)	Medical	89 (27.55%)	2 (1.96%)	87 (43.07%)	0 (0.00%)	< 0.001
	Surgical	65 (20.12%)	45 (44.12%)	10 (4.95%)	10 (52.63%)	
	Intensive Care Unit	167 (51.70%)	54 (52.94%)	104 (51.49%)	9 (47.37%)	
	Missing	2 (0.62%)	1 (0.98%)	1 (0.50%)	0 (0.00%)	
Culture results	No growth	142 (43.96%)	41 (40.20%)	92 (45.54%)	9 (47.37%)	0.576
	Gram-positive Cocci	88 (27.24%)	24 (23.53%)	59 (29.21%)	5 (26.32%)	
	Gram-negative Bacilli	82 (25.39%)	33 (32.35%)	45 (22.28%)	4 (21.05%)	
	Yeast Cells	10 (3.10%)	3 (2.94%)	6 (2.97%)	1 (5.26%)	
	Missing	1 (0.31%)	1 (0.98%)	0 (0.00%)	0 (0.00%)	
Indications for MRSA coverage	Immunosuppression or comorbidities	150 (46.44%)	64 (62.75%)	79 (39.11%)	7 (36.84%)	< 0.001
	Admission last for 90 days	70 (21.67%)	31 (30.39%)	30 (14.85%)	9 (47.37%)	
	Severe infection	37 (11.46%)	1 (0.98%)	33 (16.34%)	3 (15.79%)	
	IV antibiotics last for 90 days	12 (3.72%)	2 (1.96%)	10 (4.95%)	0 (0.00%)	
	Post-hemodialysis	1 (0.31%)	0 (0.00%)	1 (0.50%)	0 (0.00%)	
	Not documented/No clear indications	53 (16.41%)	4 (3.92%)	49 (24.26%)	0 (0.00%)	
Duration, days	Median (IQR)	5 (3–8)	5 (3–6)	5 (3–8)	6 (3–9)	0.448
Infection Type	Bloodstream / sepsis-related infections	146 (45.20%)	48 (47.06%)	91 (45.05%)	7 (36.84%)	0.486
	Respiratory tract infections	116 (35.91%)	39 (38.24%)	66 (32.67%)	11 (57.89%)	
	Central nervous system infections	5 (1.55%)	0 (0.00%)	5 (2.48%)	0 (0.00%)	
	Skin and soft tissue infections	29 (8.98%)	8 (7.84%)	21 (10.40%)	0 (0.00%)	
	Other healthcare-associated	1 (0.31%)	0 (0.00%)	1 (0.50%)	0 (0.00%)	
	No documented infection	26 (8.05%)	7 (6.86%)	18 (8.91%)	1 (5.26%)	

Missing categories reflect unavailable source data. Abbreviations: ICU, intensive care unit; MRSA, methicillin-resistant *Staphylococcus aureus*; BMI, body mass index; IQR, interquartile range. Culture results were categorized according to the predominant microbiological finding documented for each patient. When more than one organism category was reported, the clinically most relevant culture result was used for tabulation

### Clinical outcomes

Overall, in-hospital mortality was 126 (39.01%), and clinical improvement was 168 (52.01%). Across treatment groups, in-hospital mortality was numerically highest in the patients who received empirical teicoplanin (57.89%) versus the linezolid group (42.57%) and the vancomycin group (35.82%), but this difference did not reach statistical significance (*p* = 0.121). Clinical improvement rates were broadly comparable (52.94% linezolid, 53.47% vancomycin, 31.58% teicoplanin; *p* = 0.312). Length of stay was longest in the teicoplanin group (median 30 days, IQR 11–60) compared with linezolid and vancomycin (16 days, IQR 9–30 for vancomycin; 14 days, IQR 9–30 for linezolid), though differences were not significant (*p* = 0.629), as shown in Table 2.

Most patients experienced no reported side effects, but patients who received vancomycin had higher recorded events (AKI 3.00%, elevated liver enzymes 2.50%) relative to the linezolid group (AKI 0.99%, no liver enzyme elevations) and the teicoplanin group (no AKI, one gastrointestinal upset) (*p* = 0.002). Among laboratory/clinical response markers, only CRP change differed significantly between groups (*p* = 0.009), with median reductions with linezolid (–378.46) and vancomycin (–290.83), contrasted by a rise with teicoplanin (+ 507.52). Changes in temperature, creatinine clearance, ALT, AST, white blood cell count, and platelet count showed no significant between-group differences (all *p* > 0.05).

**Table 2:** Comparison of clinical outcomes by treatment group

		Linezolid	Vancomycin	Teicoplanin	*P*-value
In-hospital Mortality, *n* (%)		43 (42.57%)	72 (35.82%)	11 (57.89%)	0.121
Clinical improvement, n (%)		54 (52.94%)	108 (53.47%)	6 (31.58%)	0.312
Documented Side effects	No	100 (99.01%)	189 (94.50%)	18 (94.74%)	0.002*
	AKI	1 (0.99%)	6 (3.00%)	0 (0.00%)	
	GI Upset	0 (0.00%)	0 (0.00%)	1 (0.99%)	
	Elevated Liver Enzymes	0 (0.00%)	5 (2.50)	0 (0.00%)	
Length of hospital Stay	Median (IQR)	14 (9–30)	16 (9–30)	30 (11–60)	0.629
Clinical and laboratory parameters	Change in temperature	-0.16 (-0.27 to -0.06)	-0.26 (-0.36 to -0.16)	-0.08 (-0.08 to 0.22)	0.281
	Change in CrCl	7.22 (2.67 to 11.77)	5.68 (1.41 to 9.95)	-0.45 (-10.81 to 9.92)	0.558
	Change in ALT	53.22 (-20.52 to 126.98)	60.54 (-1.73 to 122.81)	-74.89 (-426.92 to 277.15)	0.448
	Change in AST	79.12 (-54.73 to 212.98)	93.91 (-21.43 to 209.28)	39.88 (-119.67 to 199.43)	0.951
	Change in CRP	-378.46 (-590.51 to -166.40)^a^	-290.83 (-425.54 to -156.12)^a^	507.52 (-688.92 to 1703.98)^b^	0.009
	Change in WBC	-2.22 (-3.85 to -0.60)	-1.29 (-2.42 to -0.166)	-1.22 (-4.62 to 2.19)	0.631
	Change in Platelet count	-35.37 (-80.97 to 10.22)	-7.76 (-25.51 to 9.98)	-10.06 (-63.31 to 43.18)	0.392

Superscripts a/b in the CRP row indicate pairwise differences between groups after post-hoc Tukey testing (different letters = significantly different; same letters = not significantly different). Abbreviations: AKI, acute kidney injury; CrCl, creatinine clearance; ALT, alanine aminotransferase; AST, aspartate aminotransferase; CRP, C-reactive protein; WBC, white blood cell count; IQR, interquartile range

### Risk factors of in-hospital mortality

In univariate analyses, non-survivors were significantly older than survivors (76.96 ± 13.85 vs. 67.03 ± 18.17 years; *p* < 0.001) and were more frequently admitted to the ICU (60.32% vs. 46.15%), whereas survivors were more often admitted to medical wards (33.33% vs. 19.05%; overall ward distribution *p* = 0.006). Gender, BMI, and treatment duration did not differ meaningfully between groups (all *p* > 0.05). Culture category distributions were not statistically different (*p* = 0.132), although Gram-negative bacilli were more common among non-survivors (31.75% vs. 21.03%). Indications for MRSA coverage differed (*p* < 0.001), with immunosuppression/comorbidities more frequent in non-survivors (56.35% vs. 39.49%); prior IV antibiotics were also numerically higher in non-survivors (6.35% vs. 2.05%). Non-survivors had a longer length of stay (median 22 [11–30] vs. 13 [8–30] days; *p* < 0.001), as shown in Table 3.

The infection category differed significantly between survivors and non-survivors (*p* < 0.001). Non-survivors more frequently had respiratory tract infections (45.24% vs. 30.26%) and, to a lesser extent, bloodstream/sepsis-related infections (47.62% vs. 43.08%), whereas survivors more commonly had skin and soft tissue infections (14.36% vs. 0.79%). At baseline, non-survivors had lower temperature (37.07 ± 0.44 vs. 37.26 ± 0.62 °C; *p* = 0.004), reduced renal function (CrCl 39.84 [17.73–63.72] vs. 53.15 [22.20–91.15] mL/min; *p* = 0.027), higher AST (38.00 [22.00–72.00] vs. 24.00 [16.00–38.50] U/L; *p* < 0.001), and lower platelet counts (218.00 [140.25–319.50] vs. 274.00 [192.00–355.00] ×10⁹/L; *p* = 0.002). Baseline ALT, CRP, WBC, and the median treatment duration were not significantly associated with mortality (all *p* > 0.05).

**Table 3:** Risk factors for in-hospital mortality

Risk Factors		Survivors	Non-Survivors	*P*-value
Age, years	Mean ± SD	67.03 ± 18.17	76.96 ± 13.85	< 0.001
Gender, n (%)	Male	96 (49.23%)	75 (59.52%)	0.071
	Female	99 (50.77%)	51 (40.48%)	
BMI, Kg/m^2^	Mean ± SD	28.62 ± 8.51	27.80 ± 7.11	0.734
Ward of Admission, n (%)	Medical	65 (33.33%)	24 (19.05%)	0.006
	Surgical	40 (20.51%)	24 (19.05%)	
	Intensive Care Unit	90 (46.15%)	76 (60.32%)	
	Missing	0 (0.00%)	2 (1.59%)	
Culture results	No growth	93 (47.69%)	49 (38.89%)	0.132
	Gram-positive Cocci	55 (28.21%)	32 (25.40%)	
	Gram-negative Bacilli	41 (21.03%)	41 (32.54%)	
	Yeast Cells	6 (3.08%)	4 (3.17%)	
Indications for MRSA coverage	Immunosuppression or comorbidities	77 (39.49%)	71 (56.35%)	< 0.001
	Admission last for 90 days	46 (23.59%)	24 (19.05%)	
	Severe infection	23 (11.79%)	14 (11.11%)	
	IV antibiotics last for 90 days	4 (2.05%)	8 (6.35%)	
	Post-hemodialysis	0 (0.00%)	1 (0.79%)	
	Missing	45 (23.08%)	8 (6.35%)	
Duration, days	Median (IQR)	5.00 (3.00–8.00)	5.00 (2.00–8.00)	0.476
Length of Hospital Stay	Median (IQR)	13.00 (8.00–30.00)	22.00 (11.00–30.00)	< 0.001
Infection Type	Bloodstream / sepsis-related infections	84 (43.08%)	60 (47.62%)	< 0.001
	Respiratory tract infections	59 (30.26%)	57 (45.24%)	
	Central nervous system infections	4 (2.05%)	1 (0.79%)	
	Skin and soft tissue infections	28 (14.36%)	1 (0.79%)	
	Other healthcare-associated	0 (0.00%)	1 (0.79%)	
	No documented infection	20 (10.26%)	6 (4.76%)	
Laboratory	Baseline Temperature	37.26 ± 0.62	37.07 ± 0.44	0.004
	Baseline CrCl	53.15 (22.20–91.15)	39.84 (17.73–63.72)	0.027
	Baseline ALT	22.50 (14.00–36.25)	26.00 (16.00–58.00)	0.098
	Baseline AST	24.00 (16.00–38.50)	38.00 (22.00–72.00)	< 0.001
	Baseline CRP	1131.00 (549.00–1838.00)	1072.00 (431.50–1690.75)	0.662
	Baseline WBCs	11.35 (8.17–16.00)	11.30 (8.53–17.38)	0.426
	Baseline Platelet count	274.00 (192.00–355.00)	218.00 (140.25–319.50)	0.002

ICU, intensive care unit; MRSA, methicillin-resistant *Staphylococcus aureus*; CrCl, creatinine clearance; ALT, alanine aminotransferase; AST, aspartate aminotransferase; CRP, C-reactive protein; WBC, white blood cell count

In univariate models, higher age was associated with greater in-hospital mortality (OR 1.04 per year; 95% CI 1.02–1.06; *p* < 0.001), while being < 60 years was protective versus ≥ 60 (OR 0.37; 0.20–0.71; *p* = 0.003). Male sex was not significantly associated with death (OR 1.52; 0.96–2.39; *p* = 0.072). Non-ICU admission was associated with lower mortality relative to ICU (OR 0.54; 0.34–0.86; *p* = 0.009). Length of hospital stay associated with increased in-hospital mortality (OR 1.01 per day; 1.00–1.02; *p* = 0.020). Among baseline labs, higher temperature was associated with lower mortality (OR 0.50 per °C; 0.31–0.81; *p* = 0.005), higher creatinine clearance with lower mortality (OR 0.993 per mL/min; 0.987–0.999; *p* = 0.027), and higher platelet count with lower mortality (OR 0.998 per 10⁹/L; 0.996–0.999; *p* = 0.016). BMI, culture result, MRSA-coverage indications, treatment duration, ALT, AST, CRP, and WBC were not significant (all *p* > 0.05).

In multivariable analysis, independent predictors of in-hospital mortality were older age (OR 1.05 per year; 1.02–1.08; *p* < 0.001), male sex (OR 1.81; 1.05–3.13; *p* = 0.032), ICU admission (non-ICU vs. ICU: OR 0.45; 0.26–0.78; *p* = 0.004, indicating lower risk outside ICU), and longer length of stay (OR 1.009 per day; 1.001–1.016; *p* = 0.023). The protective association of higher baseline temperature and the adverse associations with lower creatinine clearance and lower platelets attenuated and were no longer statistically significant after adjustment (all *p* > 0.05). Age group, BMI metrics, culture result, and MRSA-coverage indications did not remain independently associated with mortality, as shown in Table 4.

**Table 4:** Univariate and multivariable logistic regression for in-hospital mortality

Variables		Univariate regression		Multivariate Regression	
		OR (95% CI)	*P*-value	OR (95% CI)	*P*-value
Age, years		1.04 (1.02–1.06)	< 0.001	1.05 (1.02–1.08)	< 0.001
Age group	< 60	0.37 (0.20–0.71)	0.003	1.52 (0.48–4.79)	0.471
	≥ 60	Reference			
Gender, n (%)	Male	1.52 (0.96–2.39)	0.072	1.81 (1.05–3.13)	0.032
	Female	Reference			
BMI, Kg/m^2^		0.98 (0.95–1.02)	0.404	-	
BMI Category	< 30	1.65 (0.98–2.79)	0.060	-	
	≥ 30	Reference			
ICU Admission	Non-ICU	0.54 (0.34–0.86)	0.009	0.45 (0.26–0.78)	0.004
	ICU	Reference			
Culture results	Gram-negative Bacilli	1.67 (0.91–3.11)	0.100	-	
	Gram-positive Cocci	Reference			
Indications for MRSA coverage	Immunosuppression or comorbidities	Reference			
	Admission last for 90 days	0.56 (0.31–1.02)	0.058	-	
	Severe infection	0.66 (0.32–1.38)	0.270	-	
	IV antibiotics last for 90 days	2.17 (0.63–7.51)	0.222	-	
	Post-hemodialysis	NE			
Duration, days		0.98 (0.95–1.03)	0.502	-	
Length of Hospital Stay, days		1.01 (1.00–1.02)	0.020	1.009 (1.001–1.016)	0.023
Laboratory	Baseline Temperature	0.500 (0.310–0.810)	0.005	0.728 (0.428–1.237)	0.241
	Baseline CrCl	0.993 (0.987–0.999)	0.027	0.998 (0.991–1.005)	0.513
	Baseline ALT	0.999 (0.998–1.001)	0.903	-	
	Baseline AST	1.002 (0.999–1.004)	0.103	-	
	Baseline CRP	0.999 (0.999–1.000)	0.511	-	
	Baseline WBCs	1.016 (0.985–1.048)	0.292	-	
	Baseline Platelet count	0.998 (0.996–0.999)	0.016	0.999 (0.997–1.001)	0.285

Odds ratios (ORs) with 95% confidence intervals (CIs) from logistic regression models. Multivariable model includes covariates retained after adjustment (see columns). Continuous predictors are per unit increase: age (years), BMI (kg/m²), length of stay (days), temperature (°C), creatinine clearance (CrCl; mL/min), alanine aminotransferase (ALT; U/L), aspartate aminotransferase (AST; U/L), C-reactive protein (CRP; mg/L), white blood cells (WBC; ×10⁹/L), platelet count (×10⁹/L). NE = not estimable. P-values < 0.05 are considered statistically significant

**Fig. 1:**
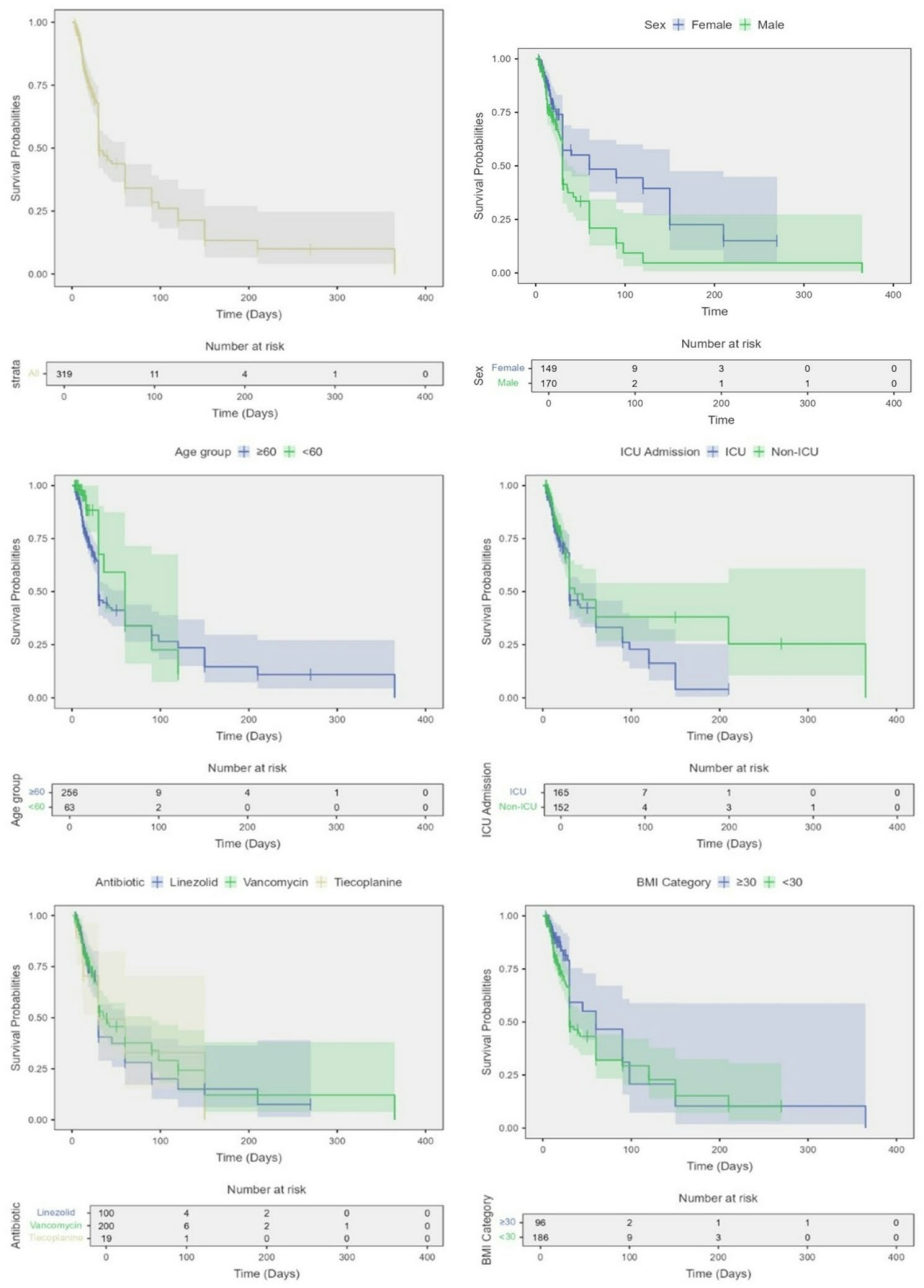
Kaplan–Meier survival curves for time to in-hospital mortality overall and by key subgroups; Kaplan–Meier estimates of survival probability from the start of anti-MRSA therapy to in-hospital death (event) or discharge/censoring. Shaded bands denote 95% confidence intervals; vertical ticks indicate censored observations. Number-at-risk tables are shown beneath each panel. (**a**) Overall cohort. (**b**) Sex (female vs. male). (**c**) Age group (≥ 60 vs. < 60 years). (**d**) Admission location (ICU vs. non-ICU). (**e**) Initial antibiotic (linezolid, vancomycin, teicoplanin). (**f**) BMI category (≥ 30 vs. < 30 kg/m²)

In univariate Cox models, male sex was associated with a significantly higher hazard of in-hospital mortality (HR 1.78, 95% CI 1.23–2.56, *p* = 0.002). No other covariate reached significance: age < 60 years showed a non-significant lower hazard versus ≥ 60 (HR 0.65, 0.37–1.13, *p* = 0.124); BMI < 30 versus ≥ 30 (HR 1.39, 0.90–2.14, *p* = 0.137); non-ICU versus ICU admission (HR 0.77, 0.53–1.11, *p* = 0.165); Gram-negative bacilli versus Gram-positive cocci (HR 1.05, 0.65–1.68, *p* = 0.855); and antibiotic choice compared with linezolid (vancomycin: HR 0.85, 0.58–1.25, *p* = 0.470; teicoplanin: HR 0.99, 0.51–1.92, *p* = 0.971), as shown in Table 5**and** Fig. 1.

**Table 5:** Univariate cox proportional hazards for time to in-hospital mortality

Variables		HR (95% CI)	*P*-value
Age group	< 60	0.65 (0.37–1.13)	0.124
	≥ 60	Reference	
Gender	Male	1.78 (1.23–2.56)	0.002
	Female	Reference	
BMI Category	< 30	1.39 (0.90–2.14)	0.137
	≥ 30	Reference	
ICU Admission	Non-ICU	0.77 (0.53–1.11)	0.165
	ICU	Reference	
Bacteria	Gram negative Bacilli	1.05 (0.65–1.68)	0.855
	Gram-positive Cocci	Reference	
Antibiotic	Vancomycin	0.85 (0.58–1.25)	0.470
	Teicoplanin	0.99 (0.51–1.92)	0.971
	Linezolid	Reference	

Hazard ratios (HRs) with 95% confidence intervals (CIs) from univariate Cox models assessing time to in-hospital mortality. HR > 1 indicates higher hazard (faster time to death) relative to the reference; HR < 1 indicates lower hazard. P-values < 0.05 are considered statistically significant. Abbreviations: HR, hazard ratio; CI, confidence interval; ICU, intensive care unit

## Discussion

This single-center, retrospective study provides a real-world snapshot of the use and clinical outcomes of empirical anti-MRSA therapy in a hospitalized adult cohort in Saudi Arabia. The primary finding indicates that while there were no statistically significant differences in in-hospital mortality or clinical improvement rates among patients treated with vancomycin, linezolid, or teicoplanin, there were notable variations in patient selection, safety profiles, and inflammatory marker response. The high overall in-hospital mortality rate of 39% highlights the severity of illness in this cohort, a finding consistent with other regional studies of severe MRSA infections [15].

The absence of a statistically significant difference in mortality and clinical improvement between the three antibiotic groups is a central finding of this study. This observation is largely consistent with the broader body of international evidence [16, 17]. For instance, a recent comprehensive meta-analysis found comparable all-cause mortality and clinical cure rates between linezolid and glycopeptides like vancomycin and teicoplanin for MRSA bacteremia [16]. Another meta-analysis also reported no significant differences in mortality for nosocomial pneumonia when comparing linezolid with glycopeptides [17]. Our results, derived from a real-world Saudi cohort, appear to reinforce this general consensus of therapeutic equivalence in terms of primary efficacy outcomes; however, it is crucial to note that some literature suggests superiority for linezolid in specific scenarios, such as improved clinical cure and microbiological eradication in MRSA pneumonia, even if this does not translate to a mortality benefit [18].

A particularly noteworthy finding was the divergent response in CRP levels, where linezolid and vancomycin were associated with a significant reduction, whereas teicoplanin was associated with an increase. This finding, coupled with the numerically highest mortality rate (57.9%) and lowest clinical improvement rate (31.6%) in the teicoplanin group, suggests a potential lack of efficacy in the patients who received it. However, this must be interpreted with extreme caution. The teicoplanin group was very small (*n* = 19) and had a significantly higher proportion of patients with COVID-19 pneumonia (47.4%), a condition associated with severe inflammatory dysregulation and high mortality independent of bacterial co-infection. International studies comparing teicoplanin and vancomycin have yielded mixed results; while some find comparable efficacy [19], others report higher clinical failure or mortality with teicoplanin in specific contexts like pneumonia [20]. A network meta-analysis even found teicoplanin to be inferior to linezolid for treating pneumonia [21]. Therefore, our findings regarding teicoplanin are more likely a reflection of confounding by indication and small sample size rather than a true measure of drug inferiority.

Our study corroborates established safety concerns associated with these agents. The higher incidence of AKI recorded in the vancomycin group (3.0%) compared to linezolid (1.0%) and teicoplanin (0%) aligns with extensive literature identifying vancomycin as a significant cause of nephrotoxicity. Multiple meta-analyses have confirmed a lower risk of renal dysfunction with linezolid and teicoplanin compared to vancomycin [18, 19], a finding that has positioned them as important alternatives, especially in patients with pre-existing renal impairment or those receiving concomitant nephrotoxins. While our study did not capture thrombocytopenia, other systematic reviews have highlighted this as a key adverse event associated with linezolid, often leading to treatment discontinuation [22, 23], which contrasts with the better hematological safety profile of glycopeptides.

An interesting observation was that 44% of patients receiving empirical anti-MRSA therapy had no microbial growth on culture. This highlights a significant challenge in antimicrobial stewardship; the difficulty in distinguishing bacterial infection, particularly MRSA, from non-infectious inflammatory states in critically ill patients. This practice of broad empirical coverage in the absence of microbiological confirmation contributes to antibiotic overuse, selection pressure for resistance, and potential for drug-related toxicity [15]. This finding is particularly pertinent in the context of the COVID-19 pandemic, during which many of our patients were treated and where bacterial co-infection rates were often lower than initially presumed [24]. This underscores a critical need for enhanced diagnostic stewardship, including the use of rapid diagnostic tests and biomarkers, to guide more targeted de-escalation of therapy.

The regression analysis robustly identified older age, male sex, ICU admission, and a longer hospital stay as independent predictors of in-hospital mortality. These findings are consistent with a large body of literature on outcomes in sepsis and critical illness and reflect the overwhelming impact of host factors and disease severity on patient survival [25]. The association of male sex with higher mortality has been noted in various infectious diseases, including sepsis and COVID-19 [26]. The fact that the choice of antibiotic was not an independent predictor of mortality in our adjusted model further strengthens the interpretation that in this heterogeneous, high-risk population, patient-intrinsic factors and overall severity of illness are the primary determinants of outcome. A retrospective study from Lebanon and Saudi Arabia similarly reported a high in-hospital mortality rate of 30% in patients with MRSA pneumonia, attributing poor outcomes to disease severity and suboptimal initial therapy [15].

### Study limitations

This study has several important limitations. First, its retrospective single-center design is inherently subject to selection bias, residual confounding, and confounding by indication, particularly given the baseline differences between treatment groups. Second, no formal a priori sample size calculation was performed because all eligible consecutive patients within the predefined study period were included. Accordingly, the study may have been underpowered to detect clinically meaningful differences between treatment groups, particularly for the small teicoplanin arm, and the absence of statistically significant differences should be interpreted cautiously in view of the potential for type II error. Third, the single-center setting may limit the generalizability of the findings to other institutions or practice settings. Fourth, follow-up was limited to the index hospitalization, and 30-day or 90-day mortality could not be assessed because post-discharge outcome data were not available in this retrospective dataset. Another important limitation is that validated severity and comorbidity indices, including APACHE II, SOFA, and the Charlson Comorbidity Index, were not available in a standardized form for retrospective extraction across the full cohort. Finally, the minimum exposure threshold of 24 h may not have reflected fully optimized pharmacologic exposure for all anti-MRSA agents, particularly vancomycin and teicoplanin; therefore, some early treatment courses may have been susceptible to exposure misclassification.

## Conclusion

In this single-center, real-world cohort of hospitalized patients in Saudi Arabia receiving empirical anti-MRSA therapy, no statistically significant differences in mortality or clinical improvement were observed between linezolid, vancomycin, and teicoplanin; however, these findings should be interpreted cautiously given the observational design and limited sample size for some treatment groups. Patient outcomes were primarily driven by underlying factors such as age and severity of illness. Vancomycin was associated with a higher incidence of reported AKI, consistent with its known safety profile. The high proportion of patients treated empirically without microbiological confirmation highlights a critical need for improved diagnostic and antimicrobial stewardship. While providing valuable local data, the study’s retrospective nature and significant confounding necessitate cautious interpretation of the findings. Future prospective, randomized trials are essential to guide optimal MRSA treatment strategies in the region.

## Data Availability

The datasets used and/or analyzed during the current study are available from the corresponding author on reasonable request.

## Abbreviations

AKI: Acute kidney injury
ALT: Alanine aminotransferase
AST: Aspartate aminotransferase
BMI: Body mass index
CI: Confidence Interval
COVID-19: Coronavirus Disease 2019
CrCl: Creatinine clearance
CRP: C-reactive protein
HA-MRSA: Hospital-acquired methicillin-resistant staphylococcus aureus
HR: Hazard ratio
ICU: Intensive Care Unit
IQR: Interquartile range
IV: Intravenous
MRSA: Methicillin-resistant Staphylococcus aureus
NE: Not estimable
OR: Odds ratio
SD: Standard deviation
STROBE: Strengthening the Reporting of Observational Studies in Epidemiology
WBC: White blood cell count
